# Silent Cries, Intensify the Pain of the Life That Is Ending: The COVID-19 Is Robbing Families of the Chance to Say a Final Goodbye

**DOI:** 10.3389/fpsyt.2020.570773

**Published:** 2020-09-11

**Authors:** Jucier Gonçalves Júnior, Marcial Moreno Moreira, Modesto Leite Rolim Neto

**Affiliations:** ^1^ Departament of Internal Medicine, Santa Casa de Misericórdia de Fortaleza, Fortaleza, Brazil; ^2^ Faculty of Medicine, Federal University of Cariri, Ceara, Brazil

**Keywords:** coronavirus infection, mental health, death, pain, public health

## Introduction

The process of death and dying, despite being inherent to life, are phenomena that cause anxiety, fear, and anguish (1), with people’s reactions to this process closely correlated with their personal beliefs, social, philosophical aspects and their culture (2). During the pandemic of the new Coronavirus, COVID-19, started in Wuhan, China 668.073 deaths have already been caused in 216 countries (3). Thus, the discussion about the process of death and dying has become a daily occurrence from the news to the families but people cannot say goodbye, as well as performing their mourning rituals. This makes the process of accepting death/dying more complicated and increases the psychological suffering of those who have lost their relatives/friends. This paper aims to reflect the impact of the absence of standard rituals on the death/dying process in the context of the COVID-19 pandemic.

## Discussion

In prehistory, Neanderthals already practiced funeral rituals, being the first men to perform them through their beliefs in the idea that death was not characterized as the end, but as a transition from the world of the living to the spiritual kingdom. In ancient period, funerary rites were being improved and followed the advance of civilizations, quite prominent in ancient Egypt and the Chinese empire. They gained notoriety in the Iberian Peninsula when they were introduced by the Arabs in the 8th century (4). Therefore, there are archaeological records on funeral ritual practices since prehistory, suggesting that the concern with finitude was born concomitant with the conscience of the human being as an individual and of his life in collectivity, through ritualized care for death (5). In the 21st century, many victims of COVID-19 are dying in hospital isolation, without the presence of family or friends. The dead cannot be buried in their finest and favorite clothes. Instead, it is the grim anonymity of a hospital gown.

According to Elisabeth Kübler-Ross, in her Psychological Theory, when a person is faced with the imminence of finitude, they would experience five steps - denial, when they refuse the diagnosis; anger or revolt; negotiation or bargaining; depression, mourning process for the loss of life and, finally, acceptance of one’s finitude (6). However, rituals must be present for there to be a healthy mourning process and development. In all societies, in the event of someone’s death, the family and its social circle respond in a structured manner based on the meanings shared by the group (7). Thereby, there is no death without death rites. Rites are indispensable ways to express and solidify bonds, to encourage the sharing of emotions, to value certain situations, to ensure and reinforce social cohesion (8). Thus it is essential that family members and friends assist the death process to have the experience of elaborating the grief. In the COVID-19 pandemic, due to the high infectivity rate of the disease (9), this reality is being stolen from societies.

According to the literature, rituals go beyond action and are full of symbols. These symbols can have several meanings and make it possible to describe what we cannot express in words (5). Thus, they begin with agony and coincide with the initial phase of mourning. The segment takes place with the wake, funerals, condolences and public mourning (for prominent people), social (as in the case of the use of specific color of clothing) and psychological (the feeling of loss), extending with the cult of the dead or the visit to the cemetery, as it occurs on the day of the dead (10). Hence, in COVID-19 pandemic, the death is not a day worth living. It is a strange thing. Hospital morgues are flooded. Bodies and stories line up, coffins wrapped in pain and homesickness are led to cremation, leading to the creation of an emotional trauma.

Funeral rituals, therefore, mark the loss of a member to family members while affirming to society the value of the life of the deceased; provide the experience of mourning according to the values ​​and cultural prerogatives in which that community is immersed; they allow reflection on the paradigm of the coexistence of life and death; they redefine the meaning of life by pointing out a resizing of the lives of those who remained (11). In addition, they serve to contextualize the experience, allowing for role changes and the transition of the life cycle. They provide the family with support for the feeling of belonging to a culture capable of providing predictable responses at a time when the shock of loss leaves them numb and disjointed (5). The absence of these rites of passage also causes pain, enhances emotional traumas, which worries, because pain due to trauma can cause immediate and long-term physical consequences such as fibromyalgia and post-traumatic stress disorder. This profound disruption of organic and cognitive functions is even more common in victims of disasters (12) and pandemics.

According to Bromberg, in another perspective, funeral rituals have a therapeutic function, as (1) they help family members and friends to admit the loss of their loved ones; (2) creates a space for reflection on death as a process incorporated into life; (3) it makes it easier for family/friends to understand the grieving process and assimilate (13). In this perspective, it can be inferred that the possibility of performing funeral rites according to their customs/beliefs is a protective factor for the mental health and emotional traumas of the population that is suffering. Studies in psychology and Neurosciences demonstrate that memories loaded with emotion must be analyzed and reframed in order to overcome a traumatic phenomenon and avoid psychological distress and emotional trauma (14).

During the pandemic, several studies have shown an alarming increase in the rates of depression, suicidal ideation, anxiety (15), insomnia in addition to negative feelings such as fear, anguish, anger, stress, sadness and loneliness (16). In the psychological aspect, it is important to observe patients with their type of psychological suffering enter in silent crises and emotional traumas because this goes against all the cultural paradigms of modern society and all these feelings combined with loneliness intensify the pain of the life that is ending. This situation can also build and increase tension and it can be triggers in patients with pre-existing psychiatric diseases.

In addition, it must be considered that the process of facing death has profound correlations with the historical moment, with the social, economic and cultural prerogatives in which one is immersed. In the Middle Ages until the mid-eighteenth century, for example, death was part of people’s daily lives, existing a close relationship between the living and the dead. The image that we had of death was shown through two main characteristics: family simplicity and its publicity, and dying in public persisted until the end of the 19th century (17). Until the end of the 18th century, death was a public and well-organized ceremony, the moment before death was familiar, and the presence of relatives and friends in the patient’s room was essential. Only in the 19th century death in the West started to separate the dead from the survivors, relating to pain and emotions shown by crying, screaming and pleading. In the 19th and 20th centuries, the rites were heavily governed by the influence of the Catholic religion (4). Therefore, rituals such as the concept of death have been reinvented over time, thinking about them reflecting their effects on people’s mental health is to think and reflect to what extent the social context influences the mental health of a population. In fact, with the technological advancement and longevity of human beings, facing premature and abrupt death seems to oppose the very “sense of normality”, while challenging those who lost their loved ones to reinvent themselves in a short period of time and, in parallel, fear for their deaths, those of other loved ones and of the very society in which they are immersed, that is, of their own concept of normal.

Thus, with scientific-technological advances in medical knowledge, society became a consumer of health care. However, death and the anguish/fear of death are still a *continuum* in people’s lives. Rituals emerge as a therapeutic tool for emotional traumas in death context, an aid to understand and elaborate the strangeness of the moment, but also reconciling the living with the process of dying. During the COVID-19 pandemic, the inability to perform these rites reinforces the painful nature of death, arouses the feeling of loss in family members, friends, spouses, amplifies and causes emotional trauma and confronts society with its own fragility, both because of the speed with which it is installed and because of the novelty in ways of conducting the death process. There is a need for a deep reflection on the psychological, anthropological, sociological, and medical point of view of the death-dying process in the context of the new SARS-CoV-2 Pandemic, seeking to reconfigure the symbolisms, signifiers and meanings that this process took from 2020 and how the absence of this process impacts people’s mental health. After all, the COVID-19 is robbing families of the chance to say a final goodbye.

## Author Contributions

MLRN, JGJ, and MMM reviewed the study protocol, read, and screened articles for inclusion. All authors contributed to the article and approved the submitted version.

## Conflict of Interest

The authors declare that the research was conducted in the absence of any commercial or financial relationships that could be construed as a potential conflict of interest.
